# Improving rice grain shape through upstream ORF editing-mediated translation regulation

**DOI:** 10.1093/plphys/kiae557

**Published:** 2024-10-19

**Authors:** Qingqing Yang, Wenjie Zhu, Xu Tang, Yuechao Wu, Guanqing Liu, Dongsheng Zhao, Qiaoquan Liu, Yong Zhang, Tao Zhang

**Affiliations:** Jiangsu Key Laboratory of Crop Genomics and Molecular Breeding/Zhongshan Biological Breeding Laboratory/Key Laboratory of Plant Functional Genomics of the Ministry of Education, Agricultural College of Yangzhou University, Yangzhou 225009, China; Jiangsu Co-Innovation Center for Modern Production Technology of Grain Crops/Jiangsu Key Laboratory of Crop Genetics and Physiology, Yangzhou University, Yangzhou 225009, China; Department of Biotechnology, School of Life Sciences and Technology, Center for Informational Biology, University of Electronic Science and Technology of China, Chengdu 610054, China; Jiangsu Key Laboratory of Crop Genomics and Molecular Breeding/Zhongshan Biological Breeding Laboratory/Key Laboratory of Plant Functional Genomics of the Ministry of Education, Agricultural College of Yangzhou University, Yangzhou 225009, China; Department of Biotechnology, School of Life Sciences and Technology, Center for Informational Biology, University of Electronic Science and Technology of China, Chengdu 610054, China; Integrative Science Center of Germplasm Creation in Western China (Chongqing) Science City, Chongqing Key Laboratory of Tree Germplasm Innovation and Utilization, School of Life Sciences, Southwest University, Chongqing 400715, China; State Key Lab of Rice Biology, China National Rice Research Institute, Hangzhou 311400, Zhejiang, China; Jiangsu Key Laboratory of Crop Genomics and Molecular Breeding/Zhongshan Biological Breeding Laboratory/Key Laboratory of Plant Functional Genomics of the Ministry of Education, Agricultural College of Yangzhou University, Yangzhou 225009, China; Jiangsu Key Laboratory of Crop Genomics and Molecular Breeding/Zhongshan Biological Breeding Laboratory/Key Laboratory of Plant Functional Genomics of the Ministry of Education, Agricultural College of Yangzhou University, Yangzhou 225009, China; Jiangsu Key Laboratory of Crop Genomics and Molecular Breeding/Zhongshan Biological Breeding Laboratory/Key Laboratory of Plant Functional Genomics of the Ministry of Education, Agricultural College of Yangzhou University, Yangzhou 225009, China; Jiangsu Key Laboratory of Crop Genomics and Molecular Breeding/Zhongshan Biological Breeding Laboratory/Key Laboratory of Plant Functional Genomics of the Ministry of Education, Agricultural College of Yangzhou University, Yangzhou 225009, China; Jiangsu Co-Innovation Center for Modern Production Technology of Grain Crops/Jiangsu Key Laboratory of Crop Genetics and Physiology, Yangzhou University, Yangzhou 225009, China; Department of Biotechnology, School of Life Sciences and Technology, Center for Informational Biology, University of Electronic Science and Technology of China, Chengdu 610054, China; Integrative Science Center of Germplasm Creation in Western China (Chongqing) Science City, Chongqing Key Laboratory of Tree Germplasm Innovation and Utilization, School of Life Sciences, Southwest University, Chongqing 400715, China; Jiangsu Key Laboratory of Crop Genomics and Molecular Breeding/Zhongshan Biological Breeding Laboratory/Key Laboratory of Plant Functional Genomics of the Ministry of Education, Agricultural College of Yangzhou University, Yangzhou 225009, China; Jiangsu Co-Innovation Center for Modern Production Technology of Grain Crops/Jiangsu Key Laboratory of Crop Genetics and Physiology, Yangzhou University, Yangzhou 225009, China

## Abstract

Upregulating gene expression by disturbing or destroying the upstream ORF of target genes is an efficient strategy for improving rice grain traits.

Dear Editor,

Rice (*Oryza sativa*) is vital for global food security and human health, influenced by its yield and quality. Grain shape is primarily determined by 3 key morphometric parameters, namely grain length, width, and thickness, which has a direct and important impact on both the yield and quality of rice, rendering it a critical consideration in rice breeding programs. Varieties with longer and narrower grains typically exhibit superior appearance quality and higher commercial value (Zhao et al. 2022).

To date, numerous genes/quantitative trait locus (QTLs) affecting rice grain shape have been identified, and gene-editing technologies also greatly promote the improvement of rice grain traits (Zhao et al. 2022). Despite this progress, many elite genes remain underutilized in breeding programs. The positive regulation of key genes like grain length and weight on chromosome 7 (*GLW7*) and grain width on chromosome 7 (*GW7*) is beneficial in breeding due to their minimal impact on other desirable agronomic traits. However, genetic knockout editing may not directly enhance grain shape or yield traits.

Combining upstream ORF (uORF) identification and gene-editing technology provides a promising strategy for positively regulating gene expression (Zhang et al. 2018; Si et al. 2020; Tang and Zhang 2023). The uORF is a primary regulatory element within the 5′-UTR, which potentially affects main ORF (mORF) translation, and lies upstream of the mORF in 18% to 57% of plant mRNAs (Zhong et al. 2023). Recent studies have demonstrated uORFs contribute to phenotypic diversity, and uORF-based genetic engineering for trait improvement has been performed in plants (Wang et al. 2024). Editing repressive uORFs has been successfully produced transgene-free materials with enhanced gene expression and improved performance in crops. Nonetheless, natural variation of uORF related to rice grain shape has not been found and identified, thus limiting the application of genes with breeding value in improving rice appearance quality.

In this study, considering the important impact of grain shape on rice appearance quality and yield traits, we focused on *GLW7* and *GW7* as regulatory targets, uORFs of which have not been identified (Wang et al. 2015, 2024; Si et al. 2016). Our goal was to develop a strategy for improving rice grain shape and yield through gene editing in molecular breeding (Fig. A). To explore this, we integrated bioinformatic analysis of Ribo-Seq data from a public database, which revealed 1 potential uORF in the 5′-UTR of *GLW7* and 2 uORFs at the 5′-UTR of *GW7* (Fig. B and F). Preliminary assessment results showed that altering ATG to AAA in *GLW7*-uorf1 reduced luciferase (LUC)/Renilla luciferase (REN) activity ratios compared with wild-type uORFs in the dual-LUC reporter system (Fig. D). Similar results were found for *GW7*-uorf1, whereas *GW7*-uORF2 had no effect on LUC/REN activity level (Fig. h). Therefore, 2 gRNAs targeted the sequences on both sides of *GLW7-*uORF1 (Fig. C; Supplementary Table S2), and another 2 gRNAs targeted the ATG start codon sites of *GW7*-uORF1 (Fig. G; Supplementary Table S2). Cas9-based uORF-KO constructs (Tang et al. 2019) were constructed and transformed into Nipponbare (*O. sativa* ssp. *japonica*; NIP) via *Agrobacterium tumefaciens*-mediated transformation (Zheng et al. 2023). Ten transgenic plants were produced, and after continuous screening from T_0_ to T_4_, 3 homozygous *uorf* mutants from independent transformants were selected for further analysis.

**Figure.**
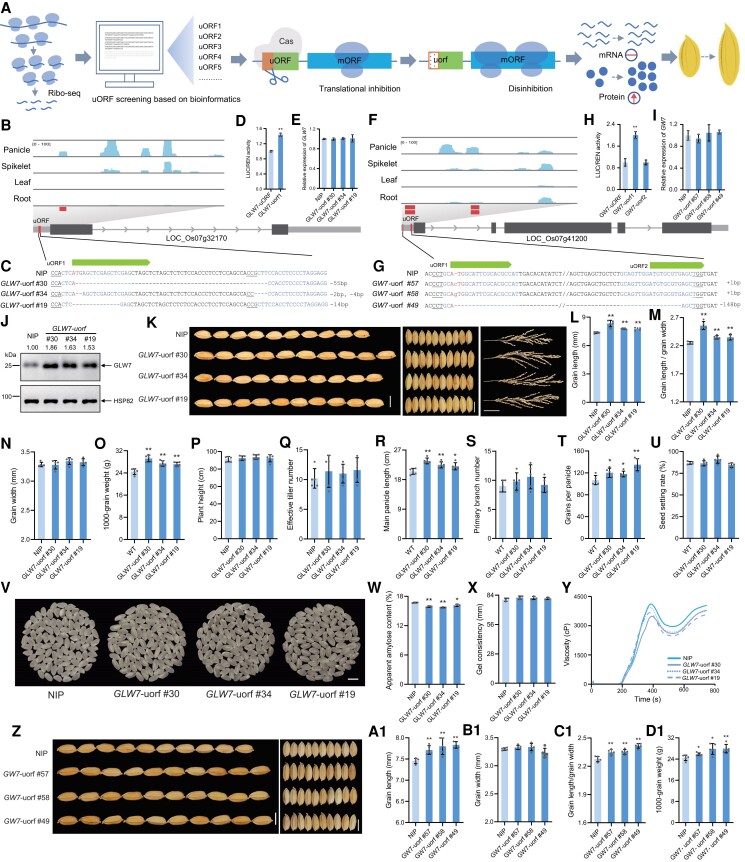
Editing uORFs of *GLW7* and *GW7* improves rice grain appearance quality. **A)** Schematic representation of improving rice grain shape by manipulating uORFs through gene editing. **B**, **F)** Schematic diagram of predicted uORFs of *GLW7* and *GW7* via Ribo-seq (http://www.ncbi.nlm.nih.gov/; PRJNA637713 and PRJNA725700) and genome sequence analysis (http://rice.plantbiology.msu.edu/pub/data/). **C**, **G)** Nucleotide variation analysis of the target sites in mutants. The target sequence is the sequence within 20-bp from the PAM (i.e. NGG) site. The ATG bases is in red. PAM is underlined. “-” means deletion. Lowercase bases means insertion. **D**, **H)** The effect of ATG mutation to AAA in the 5′ lead sequence on translation of the mORF in the dual-LUC reporter system. **E**, **I)** Expressions of *GLW7* and *GW7* at the RNA level in young panicles of wild type and uorf mutants. **J)** GLW7 level was analyzed with western blot. Rice HSP82 was used as the internal control. The gray ratios were measured using ImageJ software. **K)** Grain and panicle traits in *GLW7*-uorf mutants and wild type. Bar: 5 mm. **L** to **O)** Grain length (cm), grain width (cm), ratio of grain length to width, and 1,000-grain weight (g) of *GLW7*-uorf mutants and wild type. **P** to **U)** Analysis of main agronomic traits of both *GLW7*-uorf mutant and wild type. **V)** Polished rice of NIP and *GLW7-*uorf mutants. Bar: 5 cm. **W** to **Y)** Comparisons of apparent amylose content, gel consistency, and rapid viscosity analyzer spectra of rice flours of *GLW7*-uorf mutants and wild type. **Z)** Grain shape in *GW7*-uorf mutants and wild type. Bar: 5 mm. **A1** to **D1)** Grain length (cm), grain width (cm), ratio of grain length to width, and 1,000-grain weight of *GW7*-uorf mutants and wild type. Five independent plants were used for analysis of field agronomic traits (*n* = 5), while the remaining data from 3 biological replicates (*n* = 3). All data are presented as mean ± Sd. * and ** indicate significant differences from wild-type plants (*P < 0.05 and **P < 0.01) by using 2-tailed Student's *t*-tests.

To determine the genome-editing result, sequencing analysis revealed that all *uorf* mutants contained base insertions or deletions at the ATG site (Fig. C and G; Supplementary Fig. S1). To investigate the effects of uORFs on target gene expression and translation levels, we conducted quantitative PCR (qPCR) and western blot experiments. qPCR assays revealed no substantial difference in *GLW7* and *GW7* mRNA levels among various uORF mutants and NIP (Fig. E and I; Supplementary Fig. S2). Western blot assay showed that GLW7 levels of *GLW7*-uorf #30 (55-bp deletions), *GLW7*-uorf #34 (2- and 4-bp deletions), and *GLW7*-uorf #19 (14-bp deletions) increased by 86%, 63%, and 53%, respectively (Fig. J). Based on the transient assay, qPCR, western blot experiments, and previous research, we concluded that uORF mutations in *GLW7* or *GW7* upregulate translation level, rather than transcription level.

Further functional analysis showed that all *GLW7-uorf* mutants positively regulated grain length and the ratio of length to width without grain width compared to the wild type (Fig. K to N). The 1,000-grain weight values of *GLW7*-uorf #30, *GLW7*-uorf #34, and *GLW7*-uorf #19 lines were significantly increased by 19.41%, 12.14%, and 11.07%, respectively, relative to NIP (Fig. O). The results from different generations showed the stability of the mutant grain shape traits (Supplementary Table S1). Milled rice from the mutant line was longer than that of NIP, with no obvious differences observed in the chalkiness percentage and degree of the milled rice between *GLW7*-uorf mutants and NIP (Fig. V). *GLW7* has an important role in panicle development (Si et al. 2016), and all mutant lines exhibited consistent performance with respect to the effects on panicle traits, and panicle length and number of primary branches were significantly higher in the mutant plants than in the wild-type plants (Fig. R to S). The number of grains per panicle increased by 10.49% to 26.40% in mutants relative to NIP (Fig. T). Notably, no significant differences in plant height, seed setting rate, and effective tiller number were found among the mutants and NIP (Fig. P, Q, and U).

Interestingly, the mutant rice has lower amylose content, higher gel consistency, and softer taste than NIP (Fig. W to Y). The apparent amylose contents of *GLW7*-uorf #30, *GLW7*-uorf #34, and *GLW7*-uorf #19 lines were 3.23% to 5.82% lower, whereas gel consistencies increased by 1.67% to 2.51%, respectively (Fig. W and X). The viscosity profiles also changed (Fig. Y), suggesting a softer taste mutant rice compared to NIP. These results support that manipulation of *GLW7* uORF can effectively improve grain shape, yield, and quality traits.

We also examined the grain shape in *GW7*-uorf mutants, which exhibited increased grain length and length/width ratio, decreased grain width, and increased 1,000-grain weight compared with NIP (Fig. Z to D1). No significant differences were observed in the main agronomic traits (Supplementary Fig. S3). Moreover, both *GW7*-uorf mutants had lower chalkiness degrees than NIP, with no significant changes in apparent amylose, gel consistency, and rapid viscosity analyzer profile compared to NIP (Supplementary Fig. S4). These findings demonstrate that editing the uORF(s) of *GW7* can improve grain shape and weight without changing the eating quality. The above results also highlight the effectiveness of the method for mining potential uORFs in rapid rice breeding.

In summary, we introduce an efficient and convenient strategy for improve grain traits of rice by disturbing or destroying uORFs of target genes using bioinformatics and CRISPR gene editing. This provides a practical approach for crop breeding, utilizing positively regulated elite genes without modifying their coding sequence, and creating valuable genetic resources for improving appearance quality in production applications. It is possible to create rice accessions with excellent appearance, superior quality, and sustained yield under the background of good tasting quality via CRISPR/Cas9-based uORF mutant.

## Supplementary Material

kiae557_Supplementary_Data

## Data Availability

The datasets used and/or analyzed during the current study are available from the corresponding author upon request.

## Supplementary data

The following materials are available in the online version of this article.

**Supplementary Figure S1.** Sequencing chromatogram analysis of target sites in the wild type and mutants.

**Supplementary Figure S2.** *Actin*, *GLW7*, and *GW7* transcript levels analyzed using semiquantitative RT-PCR in young panicles from mutant and wild-type plants.

**Supplementary Figure S3.** Analysis of main agronomic traits of both *GW7*-uorf mutants and wild type.

**Supplementary Figure S4.** Comparisons of polished rice appearance, apparent amylose content, gel consistency, and rapid viscosity analyzer spectra of rice flours of *GW7*-uorf mutants and wild type.

**Supplementary Table S1.** Analysis of grain shape traits of different generations between uorf mutant and wild-type plants.

**Supplementary Table S2.** Primers used in this study.

## Dive Curated Terms

The following phenotypic, genotypic, and functional terms are of significance to the work described in this paper:
