# Supplementary material for: Improving rice grain shape through upstream ORF editing-mediated translation regulation
Source: Plant Physiol. 2024 Oct 19;197(1):kiae557. doi: 10.1093/plphys/kiae557 (PMC11663550; doi:10.1093/plphys/kiae557)
Supplement: kiae557_Supplementary_Data [file kiae557_supplementary_data.docx]

**
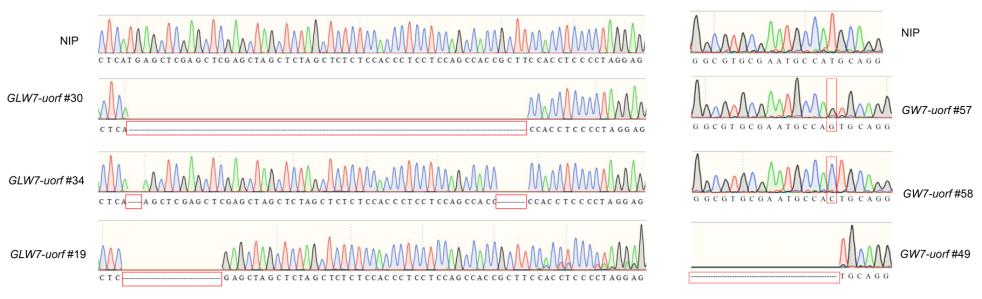
**

**Supplementary Figure S1.** Sequencing chromatogram analysis of target sites in the wild type and mutants.

**
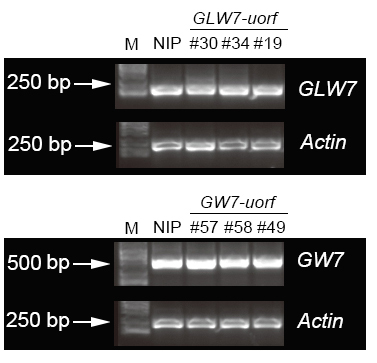
**

**Supplementary Figure S2.** *Actin*, *GLW7*, and *GW7* transcript levels analyzed using semi-quantitative RT-PCR in young panicles from mutant and wild-type plants.

**Supplementary Figure S3.** Analysis of main agronomic traits of both GW7-uorf mutants and wild type. Five independent plants were used for analysis of field agronomic traits (n=5). All data are presented as mean ± SD.

**
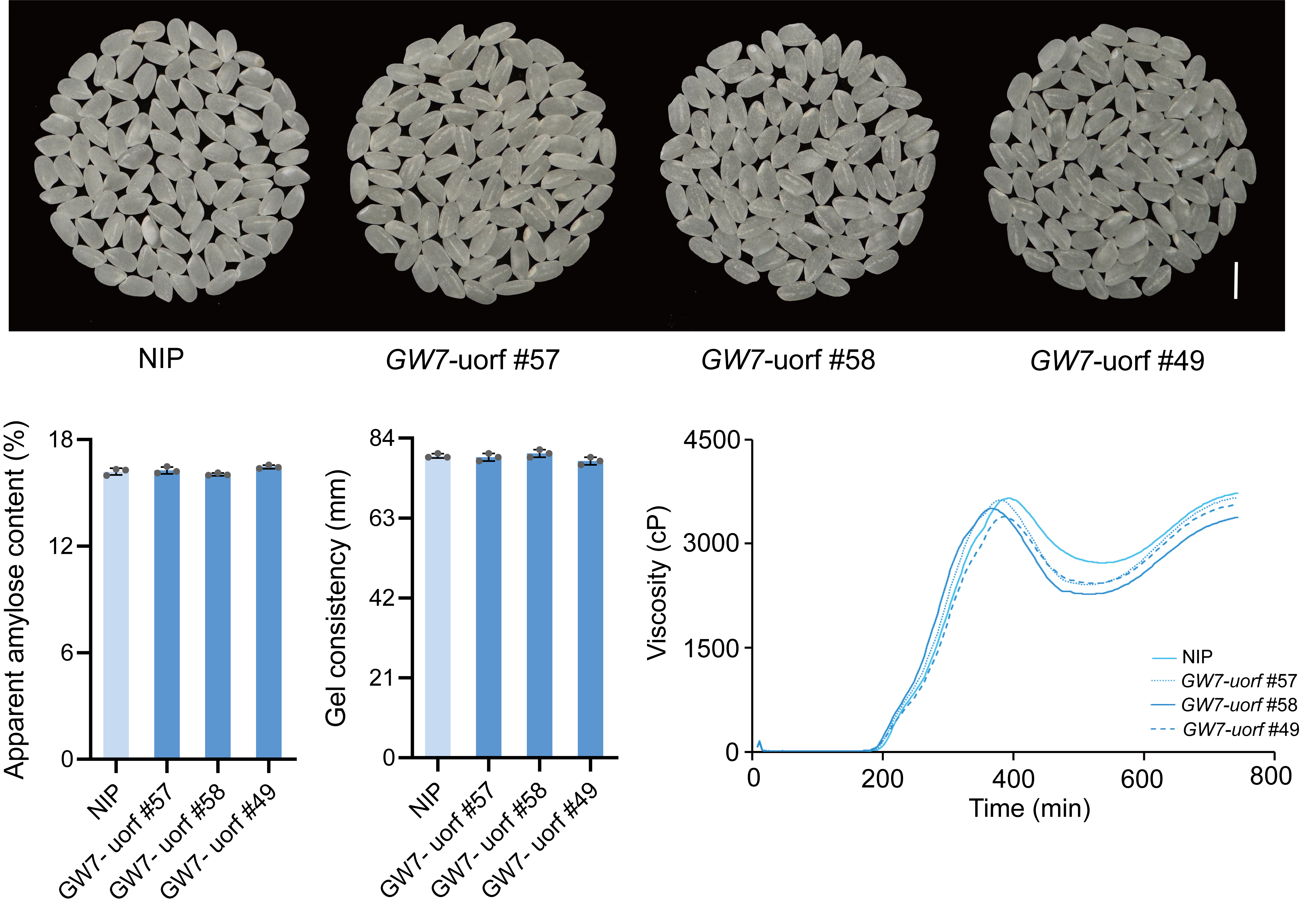
**

**Supplementary Figure S4.** Comparisons of polished rice appearance, apparent amylose content, gel consistency, and rapid viscosity analyzer spectra of rice flours of *GW7*-uorf mutants and wild type. Bar, 5 cm. Three biological replicates (n=3) were used for data analysis, the data are presented as mean ± SD.

**Supplementary Table S1. Analysis of grain shape traits of different generations between uorf mutant and wild type plants.**

| **Generation** | **Line** | **Grain shape traits** | | |
| --- | --- | --- | --- | --- |
|  |  | **Grain length (mm)** | **Grain width (mm)** | **Grain length / Grain width** |
| T3 (planted in 2022) | NIP | 7.34±0.08 | 3.06±0.03 | 2.40±0.04 |
|  | *GLW7*-uorf #30 | 7.88±0.10** | 3.09±0.03 | 2.58±0.02** |
|  | *GLW7*-uorf #34 | 7.64±0.06** | 3.09±0.03 | 2.49±0.03** |
|  | *GLW7*-uorf #19 | 7.81±0.05** | 3.08±0.02 | 2.55±0.02** |
|  | *GW7*-uorf #57 | 7.83±0.07** | 3.12±0.07 | 2.52±0.07** |
|  | *GW7*-uorf #58 | 7.64±0.12** | 3.03±0.13 | 2.55±0.08** |
|  | *GW7*-uorf #49 | 7.68±0.08** | 3.10±0.04 | 2.50±0.02** |
| T2 (planted in 2021) | NIP | 7.42±0.05 | 3.11±0.02 | 2.39±0.01 |
|  | *GLW7*-uorf #30 | 7.83±0.19** | 3.09±0.05 | 2.53±0.03** |
|  | *GLW7*-uorf #34 | 7.56±0.05** | 3.10±0.05 | 2.44±0.04* |
|  | *GLW7*-uorf #19 | 7.66±0.12** | 3.12±0.05 | 2.46±0.02** |
|  | *GW7*-uorf #57 | 7.53±0.05* | 3.10±0.03 | 2.43±0.02** |
|  | *GW7*-uorf #58 | 7.61±0.16* | 3.07±0.06 | 2.47±0.02** |
|  | *GW7*-uorf #49 | 7.69±0.06** | 3.09±0.05 | 2.49±0.04** |

The grain length and grain width from five main panicles of each transgenic line was used for statistical analysis (n=5). * and ** indicate statistical significance between transgenic and wild-type plants at *P* < 0.05 and *P* < 0.01, respectively.

**Supplementary Table S2.** **Primers used in this study.**

| **Primer name** | **Nucleotide sequence (5′→3′)** |
| --- | --- |
| sgRNA-GW7-1 | CCAGCTCACGCACATCCAACTGC |
| sgRNA-GW7-2 | ATGGCGTGCGAATGCCATGCAGG |
| sgRNA-GLW7-1 | CCTCCTAGGGGAGGTGGAAGCGG |
| sgRNA-GLW7-2 | CTCGAGCTCGAGCTCATGAGTGG |
| GLW7-jc-2F | TCCACCATCAAGACGAAACGA |
| GLW7-jc-2R | CACGGCACCCGCCTTTAT |
| GW7-jc-F | AGCAGATGCCACAGGAAG |
| GW7-jc-R | CCATCCCTCCCGCTTAT |
| GLW7-mLUC-F | ttcatttggagaggaccgcggCTCTCTCTCCCTCTCTCCTCCTCC |
| GLW7-mLUC-R | tgtttttggcgtcttccatggGGGCGGTGGCGCGCGTGA |
| GW7-mLUC-F | ttcatttggagaggaccgcggACCCTGCATGGCATTCGCACG |
| gw7-mLUC-F | ttcatttggagaggaccgcggACCCTGCAAAGCATTCGCACG |
| GW7-mLUC-R | tgtttttggcgtcttccatggCTCCTCCTCCGACTCCGACTC |
| GLW7-qF | GAGGTGCCAGGTGGAGAGGT |
| GLW7-qR | GGAACCGGCTGCATTGCT |
| GW7-qF | AAGGTCCATTCTCGCATTTCA |
| GW7-qR | AGGCAGGCTGTTCGGGTT |

**Materials and Methods**

**Plant materials, transgene constructs, and rice transformation and growth conditions**

In this study, wild-type *Oryza sativa* L. ssp. *japonica* Nipponbare (NIP) was used for transformation. The gRNA target site was selected manually, according to the sequence information of uORFs (Figure 1). To check for specificity, BLAST analyses of the gRNA target site were performed against the rice genome. The sgRNA expression cassette, including the uORF-targeting sequence, was constructed from SK-gRNA by PCR amplification with primers containing the gRNA sequence (Table S2) and then subcloned into pZHY988, a rice Cas9 editing backbone (Tang et al., 2019). Rice calli from mature embryos were used as explants for *Agrobacterium tumefaciens*-mediated transformation, according to a previously published procedure (Zheng et al., 2023).

Total genomic DNA was extracted from transformed rice leaves, as described previously (Yang et al., 2021). The targeted sequences were obtained using PCR with specific primers (Table S2), and Sanger sequencing was performed to identify mutations in the target region of uORF(s). Three or more independent mutants were screened for further analysis, and all selected transgenic lines were homozygous.

All rice materials were planted in the paddy field of Yangzhou University (Yangzhou, Jiangsu Province, China) from April to October in 2021–2023 under safety supervision for genetically modified materials and the same climate and management conditions.

**Bioinformatic analysis of Ribo-Seq data for mining potential uORFs**

The Ribo-seq raw data were sourced from two NCBI BioProjects, namely PRJNA637713 and PRJNA725700. Low-quality read filtering and sequencing adapter removal were conducted using Skewer (version 0.2.2) (Jiang et al., 2014) with the parameters “-x AGATCGGAAGAGCACACGTCTGAACTCCAGTCAC -Q 25 -l 25 -L 40” for Ribo-seq. Furthermore, ncRNA sequences for rRNA, tRNA, snoRNA, and snRNA were extracted from annotation and genome files. We then aligned the reads against ncRNA sequences using Bowtie (version 1.3.0) (Langmead et al., 2009) with the “--un” parameter to eliminate reads originating from rRNA, tRNA, snoRNA, and snRNA. The cleaned Ribo-seq reads were aligned to the rice genome (MSU7) using STAR (version 2.7.7a) (Dobin et al., 2013) with the settings “--outFilterType BySJout --alignIntronMax 200000 --outSAMtype BAM SortedByCoordinate --quantMode TranscriptomeSAM GeneCounts --outFilterMismatchNmax 2 --outFilterMultimapNmax 1 --alignEndsType EndToEnd”. Finally, Samtools (version 1.9) (Li et al., 2009) were utilized to sort and merge the aligned reads. The BAM files were visualized using IGV software (Robinson et al., 2011) to examine the distribution of reads within the uORF regions.

**Transient assay verification of uORFs in** ***Nicotiana benthamiana***

To examine the effects of predicted uORF(s), the wild-type 5′ leader sequence and artificially mutated 5′ leader sequence (ATG to AAA to prevent translation) were each cloned upstream of the luciferase (*LUC*) coding region in an expression cassette driven by the 35S promoter in pGreenII0800-LUC vector by using a dual-luciferase reporter system with two reporter genes, *LUC* and Renilla luciferase (*REN*) (Xing et al., 2020). The generated plasmids were transformed into *Agrobacterium tumefaciens* strain EHA105 for transient expression, as reported previously (Xiong et al., 2022). Briefly, *Agrobacterium tumefaciens* strains with the constructed vector were resuspended in infection buffer (10 mM MgCl_2_, 10 mM MES, pH 5.6, and 200 μM acetosyringone) to an optical density at 600 nm (0.4) and infiltrated into five-week-old *Nicotiana benthamiana* leaves. After incubation for 48 h, the infected leaves were harvested and used for measuring LUC/REN activities.

**RT-qPCR, qPCR, and western blotting**

Total RNA was extracted from young panicles of wild-type and mutated plants by using the Plant Total RNA Kit (TaKaRa, Beijing, China). The cDNAs were synthesized using Perfect Real Time PrimeScript RT reagent (TaKaRa). qPCR was performed using TB Green® Premix Ex Taq™ II (TaKaRa) and a LightCycler 480 system (Roche). The rice *actin* gene was used as the internal reference to measure the relative expression levels of rice *GLW7* and *GW7*. Gene-specific primers used for RT-qPCR and qPCR are listed in Table S2.

For western blotting, total protein was isolated from young rice panicles, as described previously (Yang et al., 2021). Proteins were detected using western blotting with antibodies specific for rice GLW7 (LOC_Os07g32170), and rice HSP82 was used as the internal control. We also amplified the CDS region of *GW7* gene (LOC_Os07g41200) and sent it to the company for the preparation of specific antibodies of GW7, but failed after two attempts. If possible, we will continue to acquire GW7 antibodies.

**Trait and grain component analyses**

The main agronomic traits, such as plant height, main panicle length, and panicle number per plant, were investigated at maturity. At least 50 fully filled grains from each panicle of a rice plant were used to measure grain length, grain width, and 1000-grain weight with the grain appearance analyzer ScanMaker (Microtek, China) and SC-E software (Microtek, China). The grain shape data for each line were derived from the average of 5 panicles.

Rice preparation and subsequent general quality measurements, such as apparent amylose content, gel consistency, and rapid viscosity analyzer profile, were performed according to a previous study (Zhang et al., 2021).

**Statistical analysis**

All data are presented as mean ± standard deviation (SD). Comparison of multiple transgenic and wild-type plants was performed using two-tailed Student’s *t*-tests. * and ** indicate statistical significance between transgenic and wild-type plants at *P* < 0.05 and *P* < 0.01, respectively.

**References**

Dobin, A., Davis, C.A., Schlesinger, F., Drenkow, J., Zaleski, C., Jha, S., Batut, P., Chaisson, M., Gingeras, T.R. (2013) STAR: ultrafast universal RNA-seq aligner. Bioinformatics, 29: 15-21

Engler, C., Kandzia, R., Marillonnet, S. (2008) A one pot, one step, precision cloning method with high throughput capability. PLoS One. 3,7.

Jiang, H.S., Lei, R., Ding, S.W., Zhu, S.F. (2014) Skewer: a fast and accurate adapter trimmer for next-generation sequencing paired-end reads. Bmc Bioinformatics, 15:182.

Langmead, B., Trapnell, C., Pop, M., Salzberg, S.L. (2009) Ultrafast and memory-efficient alignment of short DNA sequences to the human genome. Genome Biology, 10:R25.

Li, H., Handsaker, B., Wysoker, A., Fennell, T., Ruan, J., Homer, N., Marth, G., Abecasis, G., Durbin, R., Genome Project Data Processing S (2009) The Sequence Alignment/Map format and SAMtools. Bioinformatics, 25: 2078-2079.

Liu, Q.Q., Zhang, J.L., Wang, Z.Y., Hong, M.M. and Gu, M.H. (1998) A highly efficient transformation system mediated by agrobacterium tumefaciens in rice (Oryza sativa L.). Acta Phytophysiologica Sinica, 24, 259-271.

Robinson, J.T., Thorvaldsdottir, H., Winckler, W., Guttman, M., Lander, E.S., Getz, G., Mesirov, J.P. (2011) Integrative genomics viewer. Nature Biotechnology, 29: 24-26.

Tang, X., Ren, Q., Yang, L., Bao, Y., Zhong, Z., He, Y., Liu, S., Qi, C., Liu, B., Wang, Y., Sretenovic, S., Zhang, Y., Zheng, X., Zhang, T., Qi, Y. and Zhang, Y. (2019) Single transcript unit CRISPR 2.0 systems for robust Cas9 and Cas12a mediated plant genome editing. Plant Biotechnology Journal, 17, 1431-1445.

Xing, S., Chen, K., Zhu, H., Zhang, R., Zhang, H., Li, B., Gao, C. (2020) Fine-tuning sugar content in strawberry. Genome Biology, 21:230.

Xiong, M., Yu, J., Wang, J., Gao, Q., Huang, L., Chen, C., Zhang, C., Fan, X., Zhao, D., Liu, Q.Q., Li, Q.F. (2022) Brassinosteroids regulate rice seed germination through the BZR1-RAmy3D transcriptional module. Plant Physiology, 189(1), 402-418.

Yang, Q.Q., Yu, W.H., Wu, H.Y., Zhang, C.Q., Sun, S.S.M., Liu, Q.Q. (2021) Lysine biofortification in rice by modulating feedback inhibition of aspartate kinase and dihydrodipicolinate synthase. Plant Biotechnology Journal, 19(3), 490-501.

Zhang, C., Yang, Y., Chen, S., Liu, X., Zhu, J., Zhou, L., Lu, Y., Li, Q., Fan, X., Tang, S., Gu, M., Liu, Q. (2021) A rare Waxy allele coordinately improves rice eating and cooking quality and grain transparency. Journal of Integrative Plant Biology, 63(5), 889-901.

Zheng, X., Zhang, S., Liang, Y., Zhang, R., Liu, L., Qin, P., Zhang, Z., Wang, Y., Zhou, J., Tang, X. and Zhang, Y. (2023) Loss-function mutants of OsCKX gene family based on CRISPR-Cas systems revealed their diversified roles in rice. Plant Genome, e20283.
